# Supplementary material for: Multiple Administration Routes, Including Intramuscular Injection, of Oncolytic Tanapoxvirus Variants Significantly Regress Human Melanoma Xenografts in BALB/c Nude Mice Reconstituted with Splenocytes from Normal BALB/c Donors
Source: Genes (Basel). 2023 Jul 27;14(8):1533. doi: 10.3390/genes14081533 (PMC10454540; doi:10.3390/genes14081533)
Supplement: Supplementary file 1 [file genes-14-01533-s001.zip › genes-2492905-supplementary.pdf]

Supplemental Data for Genes – 2492905

Table S1. Group tumor volume averages for directly injected and non-injected melanoma xenografts in BALB/c nude mice on day 0.

**Experimental Group**	**Primary Tumor Average Volume, Day 0 (mm^3)**	**Contralateral Tumor Average Volume, Day 0 (mm^3)**
Mock Control	136.845 ± 6.856	98.095 ± 8.364
TPV/Δ66R/mIL-2, non-RC	157.121 ± 1.529	109.616 ± 8.924
TPV/Δ2L/Δ66R/FliC, non-RC	133.815 ± 4.856	84.295 ± 11.363
TPV/wt, non-RC	147.048 ± 5.831	94.651 ± 13.458
Reconstitution Control	142.739 ± 6.808	115.774 ± 15.992
TPV/Δ66R/mIL-2, RC	152.713 ± 8.831	96.579 ± 17.360
TPV/Δ2L/Δ66R/FliC, RC	147.178 ± 7.077	103.906 ± 9.305
TPV/wt, RC	153.148 ± 9.086	113.760 ± 23.48

Table S2. Group tumor volume averages for systemically treated melanoma xenografts in BALB/c nude mice on day 0.

Experimental Group	Tumor Average Volume, Day 0 (mm^3)
IM only	104.825 ± 2.957
IV only	108.216 ± 2.852
IM/IV	115.284 ± 3.375
Reconstitution Control	115.774 ± 15.992
